# Human induced pluripotent stem cells labeled with fluorescent magnetic nanoparticles for targeted imaging and hyperthermia therapy for gastric cancer

**DOI:** 10.7497/j.issn.2095-3941.2015.0040

**Published:** 2015-09

**Authors:** Chao Li, Jing Ruan, Meng Yang, Fei Pan, Guo Gao, Su Qu, You-Lan Shen, Yong-Jun Dang, Kan Wang, Wei-Lin Jin, Da-Xiang Cui

**Affiliations:** ^1^Institute of Nano Biomedicine and Engineering, Key Laboratory for Thin Film and Microfabrication of Ministry of Education, Department of Instrument Science and Engineering, National Center for Translational Medicine, Collaborative Innovational Center for System Biology, Shanghai Jiao Tong University, Shanghai 200240, China; ^2^Basic Medical Sciences Department of Biochemistry & Molecular Biology Key Laboratory of Molecular Medicine, Fudan University, Shanghai 200032, China; ^3^Department of Imaging and Nuclear Medicine, Shanghai Sixth People’s Hospital, Shanghai 20006, China

**Keywords:** Human induced pluripotent stem cell (human iPS cells), targeted imaging, hyperthermia therapy, fluorescent magnetic nanoparticles, gastric cancer, nude mice

## Abstract

**Objective:**

Human induced pluripotent stem (iPS) cells exhibit great potential for generating functional human cells for medical therapies. In this paper, we report for use of human iPS cells labeled with fluorescent magnetic nanoparticles (FMNPs) for targeted imaging and synergistic therapy of gastric cancer cells *in vivo*.

**Methods:**

Human iPS cells were prepared and cultured for 72 h. The culture medium was collected, and then was co-incubated with MGC803 cells. Cell viability was analyzed by the MTT method. FMNP-labeled human iPS cells were prepared and injected into gastric cancer-bearing nude mice. The mouse model was observed using a small-animal imaging system. The nude mice were irradiated under an external alternating magnetic field and evaluated using an infrared thermal mapping instrument. Tumor sizes were measured weekly.

**Results:**

iPS cells and the collected culture medium inhibited the growth of MGC803 cells. FMNP-labeled human iPS cells targeted and imaged gastric cancer cells *in vivo*, as well as inhibited cancer growth *in vivo* through the external magnetic field.

**Conclusion:**

FMNP-labeled human iPS cells exhibit considerable potential in applications such as targeted dual-mode imaging and synergistic therapy for early gastric cancer.

## Introduction

As stem cell nanotechnology is an emerging interesting field, theoretical and experimental studies of the interaction between nanomaterials or nanostructures and stem cells have considerably progressed^1^^-^^3^. Nanomaterials, nanostructures, and nanotechnology are important for the fundamental development of stem cell-based therapies for injuries and degenerative diseases^4^^-^^6^.

Stem cells are classified into embryonic stem cells (ESCs) and adult stem cells. ESCs comprise somatic stem cells, which are restricted to a set of lineages and normally evolves from their tissues of origin. Adult stem cells are multipotent, not pluripotent, and they can differentiate into limited cell types^7^^,^^8^. Since Evans *et al*.^9^ reported the first isolation of ESCs in 1981, stem cell research has been a novel hotspot and provides new prospects for regenerative medicine.

The application of stem cells in cancer therapy has been widely investigated because of the high totipotency and indefinite life span of these cells^8^^,^^10^. Stem cells also display intrinsic tropism in tumor sites and can spread through existing migratory pathways and non-typical routes by releasing cytokines, chemokines, and/or growth factors, which function as migration stimulatory signals^11^^-^^13^. For example, mesenchymal stem cells (MSCs) can home to sites of injury and penetrate into tumors^14^^,^^15^. In our previous study, MSCs labeled with fluorescent magnetic nanoparticles (FMNP) were used for targeted imaging and *in vivo* hyperthermia therapy for gastric cancer^16^. Studies have shown that MSCs may improve tumor growth by promoting angiogenesis, creating a niche to support the survival of cancer stem cells, suppressing immune responses against tumor cells, and/or promoting cancer metastasis^17^^,^^18^. In this regard, new cellular vehicles with tumor-homing property but without tumor growth-promoting effects must be developed. As we previously found that mES cells can target cancer cells *in vivo*^19^, we believe that hES cells can also target cancer cells *in vivo*. The pluripotent nature of hES cells enables them to differentiate into cell types of all three primary germ lineages; this characteristic presents prospects for stem cell-based therapy for human injuries and degenerative diseases^20^^,^^21^. In particular, induced pluripotent stem (iPS) cells were successfully established in 2007; somatic cells were converted into pluripotent cells by gene transfection methods, which provide potential for establishing controllable man-made stem cells^22^^,^^23^.

Currently, human iPS cells can be obtained at large scale and exhibit remarkable application prospects in drug selection, mechanism research, and organ regeneration^24^^,^^25^. Nevertheless, few studies have reported the use of human iPS cells as tumor therapeutic reagents. Human iPS cells are considered suitable for *in vivo* therapy. First, human iPS cells are effective gene delivery agents because they can deliver siRNA or drugs into tumor sites and inhibit the growth of tumor cells *in vivo*^26^^,^^27^. Second, human iPS cells can differentiate into immunological cells in tumor sites and thus kill tumor cells^28^. Finally, human iPS cells can destroy the niche of tumor cell proliferation and repress tumor growth^29^. Therefore, the development of human iPS cells for *in vivo* tumor therapy provides considerable potential for clinical translation.

In this study, we utilized the advantages of FMNPs and selected human gastric cancer as treatment target. We prepared FMNP-labeled human iPS cells and investigated their effects on gastric cancer cells *in vitro* and *in vivo*. Finally, we determined whether FMNP-labeled human iPS cells are good theranostic agents for treatment of gastric cancer or other tumor cells.

## Materials and methods

All handling and care procedures for animals were performed in accordance with the Guidelines on the Care and Use of Animals for Scientific Purposes issued by the Institutional Animal Care and Use Committee of Shanghai Jiao Tong University (No. SYXK2007-0025).

### Preparation and characterization of FMNPs

Silica-coated FMNPs were synthesized and evaluated according to our previous reports^16^^,^^30^. Ethanol (95 mL) and 2 mL of 3-aminopropyltriethoxysilane (APS) were mixed and allowed to react at room temperature for 24 h. Amino-modified FMNPs were separated using permanent magnet, washed with deionized water three times, and stored for further use. The prepared amino-modified FMNPs were characterized by a transmission electron microscope (TEM). The fluorescent and magnetic properties of amino-modified FMNPs were also determined using the photoluminescence (PL) spectra (Perkin Elmer LS 55 Spectrofluorimeter, PerkinElmer, Waltham, MA, USA) and superconducting quantum interference device magnetometer (PPMS-9 T, Quantum Design, Beijing, China), respectively. The zeta-potential value of amino-modified FMNPs was measured with particle sizing systems (NICOMP 380ZLS, PSS, Port Richey, FL, USA).

### Preparation and characterization of FMNP-labeled human iPS cells

A Cre-excisable polycistronic lentiviral vector containing *Oct4*, *Sox2*, *Lin28*, and *Nanog* genes was used to obtain iPS cells from human foreskin fibroblasts according to our previous report^16^^,^^31^. The cells were generated in a human embryonic stem (ES) cell medium consisting of DMEM/F12 (Gibco^®^, Life Technologies^TM^, USA) supplemented with Knockout SR (Gibco^®^, Life TechnologiesTM, USA), basic fibroblast growth factors (Invitrogen, USA), nonessential amino acids (Gibco^®^, USA), L-glutaMAX (Gibco^®^, USA), and β-mercaptoethanol (Gibco^®^, USA). The prepared iPS cells were identified by using our previously reported method^32^.

FMNP-labeled iPS cells were prepared by incubating human iPS cells in a culture medium containing FMNPs (50 μg/mL) for 2 h at 37 °C in 5% CO_2_. The cells were washed with phosphate-buffered saline (PBS) three times and then dissociated into single-cell suspensions by using Accumax^TM^ (Millipore^®^). Single cells were evaluated by a flow cytometer (FACSCalibur^TM^, BD Biosciences^®^, San Jose, CA) with the FL2 channel to detect FMNP-labeled cells. Acquisition data were analyzed using the FlowJo software. A fluorescence microscope (Nikon eclipse, TS100) was used to visualize the labeled iPS cell colonies stained with Prussian blue and nuclear fast red.

### Effects of FMNPs on human iPS cell viability

The effects of FMNPs on iPS cell viability were assessed using trypan blue exclusion assay. iPS cells were cultured in media containing different FMNP concentrations (0, 20, 50, and 100 μg/mL) in an incubator with humidified 5% CO_2_ and balanced air at 37 °C. The media were replaced daily. After 24, 48, and 72 h of incubation, iPS cells were washed with PBS and dissociated into single cells by using Accumax (Millipore). The number of single iPS cells was counted through trypan-blue dye exclusion technique with a hemocytometer. The number of viable (unstained) and nonviable (blue) cells were counted under a light microscope within 3 min. The viability (%) of iPS cells was calculated as follows:

$$Cell\;viability\left(\%\right)=\frac{Int_{s}}{Int_{control}}\times 100\%$$

The growth of iPS cell population was also assessed. The cells were passaged in 30 mm dishes and incubated at 37 °C under 5% CO_2_ for 1 d. The cells were then dissociated into single cells and counted using a hemocytometer. At day 1, iPS cells were treated with a complete culture medium consisting of 50 μg/mL FMNPs for 4 h, washed with the complete culture medium three times, and then cultured. At days 2, 3, and 4, the total number of iPS cells was determined daily by using a hemocytometer. Population growth curves were constructed, with untreated iPS cells as the control.

### Effects of iPS cells on the growth of gastric cancer cells

A volume of 2×10^5^ iPS cells were plated on 90 mm plates with 10 mL of the stem cell medium to obtain a conditioned medium (CM). After 72 h of incubation, CM was harvested and passed through a 0.22 μm syringe filter to remove cellular debris. The effects of CM-containing iPS cells on gastric cancer cells were evaluated by the MTT dye-reduction assay. The gastric cancer cell line MGC803 was plated at a density of 5×10^3^ cells per well on a 96-well plate with DMEM containing 10% fetal bovine serum (Invitrogen, USA). The cells were allowed to attach overnight. After 24 h, the DMEM was removed and the cells were then incubated with different CM compositions: 100% DMEM, 75% DMEM + 25% CM, 50% DMEM + 50% CM, 25% DMEM + 75% CM, 100% CM, and 100% complete culture medium. After 24, 48, and 72 h of incubation, MGC803 cells were rinsed with PBS and cell viability was assessed by the MTT dye-reduction method. Gastric cancer cell proliferation and viability were monitored using a Real-Time Cell Analyzer (RTCA) (Roche^®^). MGC803 cells were treated in media with different compositions similar to those used in the MTT assay. Electronic impedance was captured across microelectrodes, which were integrated into the bottom of the cell culture E-Plates, one time every 5 min. The curves of time-normalized cell index were plotted using RTCA software (Roche^®^).

### Preparation of nude mice model loaded with gastric cancer

Pathogen-free athymic nude (nu/nu) BALB/c mice were housed in an accredited vivarium at 22±0.5 °C and 12 h light/dark cycle. Mice were allowed access to food and water. Male athymic nude mice (4- to 6-week-old) were used to establish subcutaneous gastric cancer models. About 2×10^6^ MGC803 cells suspended in 100 μL of pure DMEM were subcutaneously injected into the right anterior flank area of each mouse. Four weeks later, tumors of approximately 5 mm in diameter were observed.

### Imaging and distribution of FMNP-labeled human iPS cells in nude mouse model

The prepared FMNP-labeled iPS cells (1×10^6^ cells) in 200 μL of PBS were injected into the tail vein of each mouse. The labeled iPS cells were also injected into mice without tumors as the negative control. Imaging of the whole animal and *ex vivo* organs was performed using *in vivo* imaging systems (IVIS-100 Imaging System, Caliper) to evaluate iPS cell distribution *in vivo*. The imaging system is equipped with a charge-coupled device camera and a red fluorescent protein (DsRed) filter (Caliper Life Sciences). At day 0, imaging was performed 5 min after FMNP-labeled iPS cells were injected into the tail vein. Other images were captured at days 1, 2, 3, 4, 7, 10, 14, and 21. The images and the fluorescent signals were acquired and analyzed using Living Image 3.2 software (Caliper Life Sciences). Red fluorescent intensity was quantified using the Image J software (National Institutes of Health, Baltimore, MD, USA), and the mean value was obtained. Magnetic resonance signals were obtained using coronal and transected T2-weighted spin echo pulse sequences, with the following imaging parameters: TR =2,500 ms, TE =80 to 90 ms, FOV =40 mm, NEX =2, and slice thickness =2.0 mm. The mice were euthanized with CO_2_. Major organs, such as the liver, heart, lung, brain, and kidney, and the tumor were collected for fluorescence imaging observation.

### Hyperthermia therapy of tumor-bearing nude mice injected with FMNP-labeled iPS cells under an external magnetic field

FMNP-labeled iPS cells were collected in a 1.5 mL Eppendorf tube and placed on the response stage of an external alternating magnetic field to estimate the hyperthermal effect. The phase of the external alternating magnetic field was changed at 63 kHz, the power was set at 7 kA/m for 10 min, and the near infrared image was recorded by FLIR^TM^ A655sc Infrared thermal mapper (FLIR^TM^, USA) and analyzed by IR Flash Professional Thermal Imaging Analysis Software (IRS software, ICI^TM^, USA). Infrared thermal images were captured at room temperature.

Mouse models of subcutaneous gastric cancer were randomly divided into three groups: the test group (10 mice, 1×10^6^ FMNP-labeled iPS cells plus external magnetic field), the control group I (10 mice, 1×10^6^ FMNP-labeled iPS cells), and the control group II (10 mice, without any treatment) to explore the potential of FMNP-labeled iPS cells in thermotherapy for gastric cancer. When the tumor size reached about 5 mm in diameter, mice in the test group and control group I were intravenously injected with FMNP-labeled iPS cells. One week after the injection, mice in the test group were placed under external alternating magnetic field four times with 63 kHz and 7 kA/m for 4 min each once a week for 1 month. Mice in the control groups I and II were reared normally. The tumor diameter in the test group, control group I, and control group II were measured weekly, and the curve of the tumor diameter was plotted.

The harvested *ex vivo* organs were treated under an external alternating magnetic field for 5 min to evaluate the hyperthermal effect of FMNP-labeled iPS cells on different organs of the tumor-free and tumor-bearing mouse models. The near infrared image of the *ex vivo* organs was recorded by FLIRTM Infrared thermal mapper. The images were analyzed and formed into a three-dimensional model by using IR Flash Professional Thermal Imaging Analysis Software (ICI, USA).

### Statistical analysis

All data were obtained from three independent experiments and presented as mean ± SD. Statistical differences were evaluated using *t* test and considered significant at the *P*<0.05 level.

## Results

### Characterization of FMNPs

**Figure S1:**
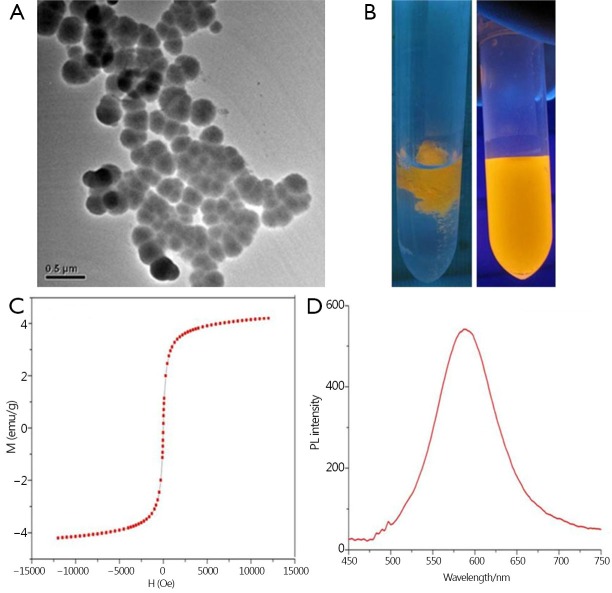
Characterization of FMNPs. (A) TEM image of amino-modified FMNPs. (B) Before and after amino-modified FMNPs in water. (C) The hysteresis curve of prepared amino-modified FMNPs. (D) The PL spectra of FMNPs. FMNPs, fluorescent magnetic nanoparticles; TEM, transmission electron microscope; PL, photoluminescence.

Similar to that in our previous reports^16^^,^^31^, the prepared FMNPs were modified with APS and encapsulated in CdTe quantum dots and Fe_3_O_4_ magnetic nanoparticles. The TEM and fluorescent images of amino-modified FMNPs, with an average diameter of 50 nm, are shown in **Figure S1A,S1B**, respectively. As shown in **Figure S1C**, the prepared amino-modified FMNPs were superparamagnetic nanoparticles composed of Fe_3_O_4_ with a saturation magnetization (Ms) value of 3.2 emu/g at room temperature. **Figure S1D** shows the PL spectra of amino-modified FMNPs, whose emission wavelength was 585 nm.

### Identification of the cultured human iPS cells

**Figure S2:**
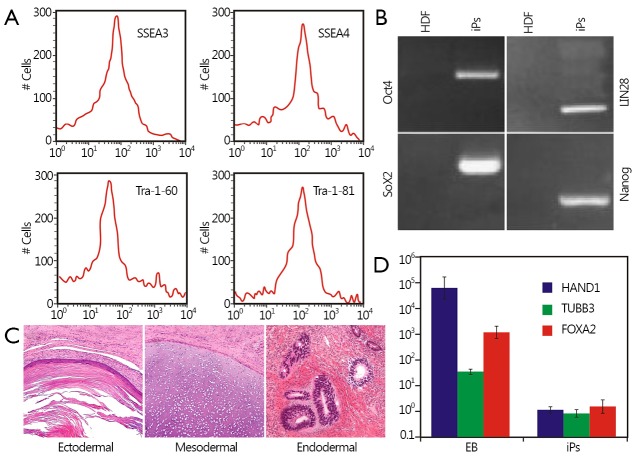
Identification of cultured human iPS cells. (A) Analysis of iPS cell-specific biomarkers expression levels by flow cytometry. (B) The expression levels of four endogenous gene *Oct4, Sox2, LIN28,* and *Nanog*. (C) H&E staining results of teratoma three germ layers (from left to right: epidermal tissue of ectoderm, mature cartilage of mesoderm, and glandular tissue of endoderm) (200×). (D) Quantitative RT-PCR analysis of three germ layers’ *HAND1, TUBB3* and *FOX-A2* gene. iPS, induced pluripotent stem.

Human iPS cells were prepared and identified according to our previous report^16^. To compare iPS and ES cells, we determined the expression levels of biomarkers specific for ES cells through fluorescent immunostaining and flow cytometry analysis. **Figure S2A** shows that ES cell-specific surface markers, such as SSEA-3, SSEA-4, Tra-1-60, and Tra-1-81, exhibited positive expression in the prepared iPS cells, whereas no positive signal was observed in control HDF cells. To identify iPS cells at the RNA level, we examined the expression levels of four genes through RT-PCR analysis. **Figure S2B** shows that endogenous Oct4, Sox2, LIN28, and Nanog were reactivated, and exogenous transgenes were silenced. This finding indicated that the pluripotent state was not maintained by continuous expression of exogenous factors. To demonstrate the pluripotency of iPS cells, we further examined teratoma growth on the back of NOD-SCID mice. H&E staining results showed that teratoma tissues comprised three germ layers (endoderm, mesoderm, and ectoderm), as shown in **Figure S2C**. The prepared iPS cells differentiated into all of the three germ layers, as evidenced by the observed neural ganglia, supporting cartilage, bone and smooth muscles, submucosa glands, and neural epithelium. To further determine the pluripotency state of iPS cells, EBs were formed after culture of iPS cells in suspension for 7 d. Quantitative RT-PCR analysis revealed that 12-d-old EBs expressed heart and neural crest derivatives-expressed 1-mesoderm, tubulin beta 3-ectoderm, and forkhead box A2-endoderm (**Figure S2D**). These results demonstrated that iPS cells were successfully prepared. See **Figures S1****,****S2** in the supplementary materials, available with the full text of this article at www.cancerbiomed.org.

### Identification and evaluation of FMNP-labeled human iPS cells

**Figure 1:**
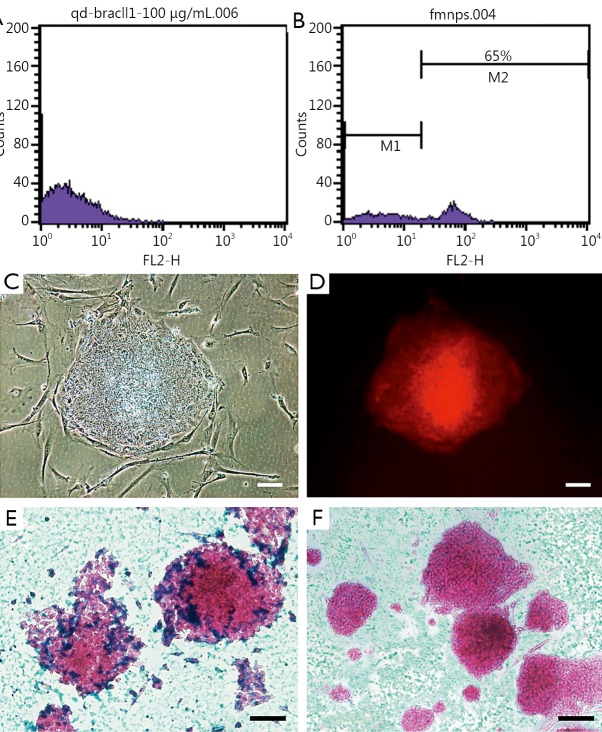
Characteristics of FMNP-labeled iPS cells. (A,B) Flow cytometry analysis results of unlabeled iPS cells and FMNP-labeled iPS cells. (C) Bright-field microscopy image of FMNP-labeled iPS cells. (D) Fluorescent microscopy image of FMNP-labeled iPS cells. (E,F) Prussian blue staining results of FMNP-labeled iPS cells and unlabeled iPS cells. (Scale bar, 50 μm). FMNP, fluorescent magnetic nanoparticle; iPS, induced pluripotent stem.

Human iPS cells were labeled with FMNPs and identified according to our previous reports^32^. The labeled iPS cells were analyzed by a flow cytometer to measure FMNP labeling efficiency in iPS cells. After incubation of FMNPs with iPS cells for 4 h, the fluorescence intensity of control unlabeled cells was determined and shown in **Figure 1A**. The labeled iPS cells exhibited strong fluorescent signals (**Figure 1B**), and 65% of iPS cells were positively stained after treatment with 50 μg/mL FMNPs for 2 h. **Figure 1C** shows the morphology of iPS cells under a bright-field microscope, and **Figure 1D** shows iPS cells with a strong red fluorescent signal under a fluorescent microscope. This finding suggested that FMNPs are located inside iPS cells. As shown in **Figure 1E**, Prussian blue staining results showed that numerous blue pellets existed around the nucleus of FMNP-labeled iPS cells. A faint blue signal was found in the unlabeled iPS control cells (**Figure 1F**). These results demonstrated that human iPS cells were successfully labeled with FMNPs.

### Effect of FMNPs on the viability of iPS cells

**Figure 2:**
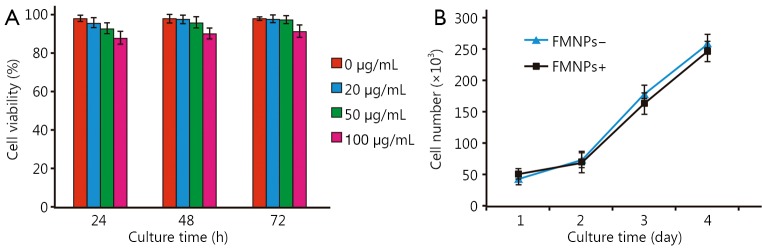
Effects of FMNPs on iPS cells. (A) Viability of iPS cells after FMNP treatment. (B) Growth rate of iPS cells after FMNP treatment. FMNP, fluorescent magnetic nanoparticle; iPS, induced pluripotent stem.

Our previous results showed that FMNPs exhibited good biocompatibility with cells at doses lower than 50 μg/mL and with mice at doses lower than 2 mg/kg body weight^33^. Nevertheless, no study has reported the effects of FMNPs on human iPS cells. **Figure 2A** shows that the mean percentage of viable iPS cells at 24, 48, and 72 h did not significantly differ between labeled and unlabeled iPS cells at FMNP doses lower than 50 μg/mL. When iPS cells were treated with 100 μg/mL FMNPs for 24 h, the viability of iPS cells reached 90%. As shown in **Figure 2B**, iPS cells incubated with FMNPs for 3 d grew well. These results showed that FMNPs exhibited good biocompatibility with human iPS cells.

### Effects of iPS cells on growth of gastric cancer cells

**Figure 3:**
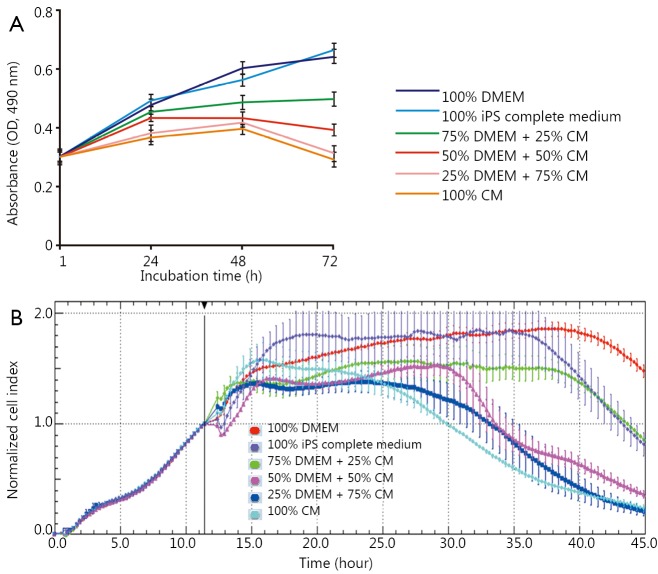
Effects of the CM from iPS cells on the gastric cancer cell line MGC803. (A,B) Inhibitory effect of different CM compositions on MGC803 cells, as revealed by MTT dye-reduction and RTCA assays. CM, conditioned medium; iPS, induced pluripotent stem; RTCA, Real-Time Cell Analyzer.

The effect of human iPS cells on gastric cancer cells is a critical parameter used to determine the suitability of using these stem cells for therapy of patients with gastric cancer. If iPS cells exhibited inhibitory effects on gastric cancer cells, then these stem cells can be used for therapy of gastric cancer *in vivo*. To investigate the feasibility of FMNP-labeled human iPS cells in inhibiting the growth of gastric cancer cells, we collected the CM from the culture bottles with iPS cells cultured for 72 h. The iPS cell complete culture medium and DMEM were used as controls. MGC803 cells were seeded into 96-well plates and continued to culture for 24 h. The media were replaced with DMEM containing different compositions and the collected CM. MTT and RTCA assays were performed at 24, 48, and 72 h. The MTT results showed that 25% and 50% diluted CM could reduce proliferation ratio of MGC803 cells after incubation for 24 h and exhibited significant inhibitory effects after 48 and 72 h of incubation (**Figure 3A**). The 75% diluted CM and 100% CM showed stronger inhibition than 50% diluted CM 24 h after the incubation. We also observed considerable growth of MGC803 cells in the complete culture medium of iPS cells and DMEM. Similarly, RTCA assay results showed that the inhibitory effects occurred 5 h after incubation with 25% diluted CM (**Figure 3B**). The proliferation of MGC803 cells weakened with increasing incubation time and CM dilution. No statistical difference existed between cells exposed to the complete culture medium of iPS cells and DMEM at 24-72 h.

Similar to our previous report^34^, the CM from iPS cells could inhibit the proliferation of MGC803 cells. This result showed that soluble factors secreted from the cultured iPS cells may play important roles in inhibiting the proliferation of gastric cancer cells. Nevertheless, the identity and mechanism of these factors, as well as their effect on the growth of MGC803 cells, remain to be elucidated.

### Fluorescent imaging and biodistribution of human iPS cells in nude mouse model

**Figure 4:**
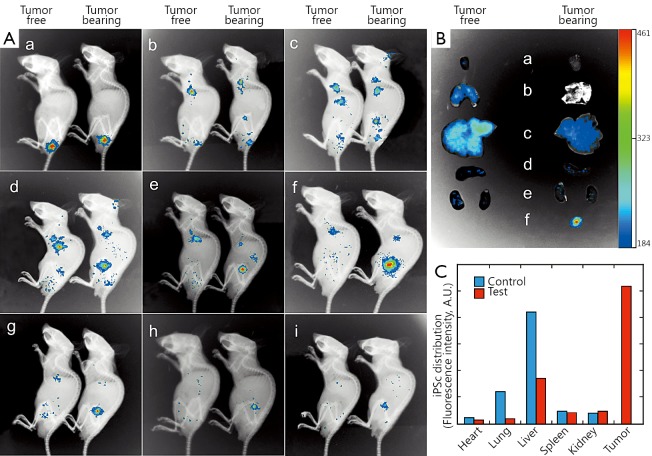
Distribution of FMNP-labeled iPS cells in the gastric cancer mouse model. (A) Whole-animal imaging using the *in vivo* imaging systems. Left: mice without tumors represent the control group (tumor-free). Right: tumor-bearing mice represent the test group. Tumor-bearing and tumor-free mice were imaged in lateral recumbent position. A_a-i_ represents imaging at day 0, 1, 2, 3, 4, 7, 10, 14, and 21, respectively. (B) Selected *ex vivo* organs were imaged to detect the fluorescent signals (on day 7 after injecting FMNP-labeled iPS cells; the highest fluorescent intensity was observed in the tumor site of tumor-bearing mice). B_a-f_ shows the organic fluorescent signals of the heart, lung, liver, spleen, kidney, and tumor, respectively. Left: the organs of the tumor-free mice. Right: the organs of tumor-bearing mice. (C) Quantified fluorescent intensity of *ex vivo* organs collected 7 d after injecting FMNP-labeled iPS cells in the tumor-free and tumor-bearing mice. FMNP, fluorescent magnetic nanoparticle; iPS, induced pluripotent stem.

We aimed to quantify distribution of intravenously injected human iPS cells *in vivo* and assess the fate of xenotransplanted human iPS cells in mice. A noninvasive *in vivo* imaging technology was employed to longitudinally monitor the distribution of FMNP-labeled iPS cells. On the first day after tail vein injection, the whole-body imaging showed that iPS cells injected into the tail vein was initially predominantly distributed in the lung region (**Figure 4A**, **Figure 4A_b_**). No evident difference was observed in iPS cell distribution *in vivo* between the tumor-free and tumor-bearing groups. On the second day following the tail vein injection, a considerable amount of iPS cells migrated from the lung to other organs (**Figure 4A_c_**). The size of FMNP signal-positive area significantly increased in the thoracic and liver regions, and the infiltration of the labeled cells was enhanced in the other parts of the mouse body. The FMNP signals began to accumulate in the tumor region of the tumor-bearing mice on the second day after the tail vein injection. The FMNP signals in the tumor regions of the tumor-bearing mice increased to the maximum at day 7. By contrast, in the tumor-free mouse group, the FMNP signals were mainly distributed in the liver (**Figure 4A_d_**) and faded from day 4 (**Figure 4A_e_**). The FMNP signals were gradually attenuated with increasing time (**Figure 4A_g-i_**), although feeble signals were still detected until day 21 (**Figure 4A_i_**). These results suggested that human iPS cells could target and track gastric cancer cells *in vivo*.

To reveal the difference in the distribution of FMNP-labeled iPS cells between the tumor-free and tumor-bearing groups, we performed *in vitro* imaging of the internal organs collected from the test mice at day 7 after the tail vein injection. Numerous FMNP-labeled iPS cells migrated and became widely distributed in liver and lung tissues of the tumor-free mice (**Figure 4B**). By contrast, in the tumor-bearing mice, the fluorescent signals significantly increased in the local gastric cancer site. A low amount of the labeled iPS cells migrated into the liver, whereas no signal was detected in the lung and heart. Our results showed that FMNP-labeled iPS cells can actively target and recognize *in vivo* gastric cancer cells.

We used ImageJ software to analyze the fluorescent intensity of the *ex vivo* organs from the test and control groups. The fluorescent signal was mainly located in the liver and lung in the control group and was localized to the tumor site in the tumor-bearing test group (**Figure 4C**). This finding provides evidence that iPS cells could actively target tumor sites *in vivo*.

### FMNP-labeled iPS cells for magnetic resonance imaging (MRI) of gastric cancer cells *in vivo*

**Figure 5:**
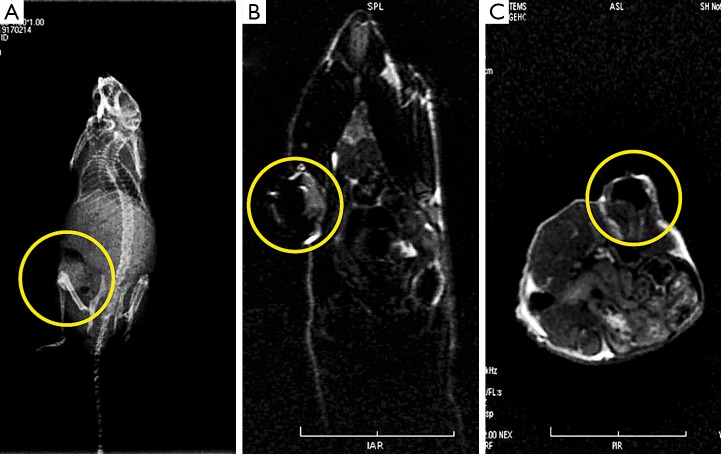
JenyX-ray and MR imaging of *in vivo* gastric cancer. (A) X-ray imaging. (B) MR T_1_ imaging. (C) MR T_2_ imaging.

As FMNP-labeled human iPS cells can be used for fluorescent imaging of *in vivo* gastric cancer cells, we performed the MRI of the FMNP-labeled human iPS cells in tumor-bearing mice. Furthermore, the ability of the labeled iPS cells to target gastric cancer cells in mouse models were monitored in the MR imaging system 7 d after the injection (**Figure 5**). FMNP-labeled iPS cells were localized to the gastric cancer site *in vivo*. **Figure 5A** shows the subcutaneous gastric cancer located in the lower left abdomen of mice. **Figures 5B****,****5C** show the MR T_1_ image (longitudinal section) and the MR T_2_ image (transverse section), respectively, of subcutaneous gastric cancer. The MR signals inside the tumors were considerably strong. Hence, FMNP-labeled iPS cells can target *in vivo* gastric cancer cells and can be used for dual-mode fluorescent and MRI imaging of *in vivo* gastric cancer.

### Effect of FMNP-labeled iPS cells on hyperthermia in gastric cancer cells *in vivo*

**Figure 6:**
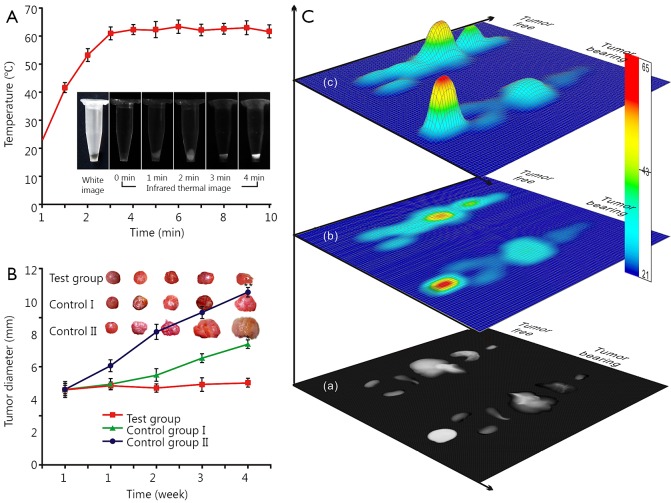
Hyperthermal effect of FMNP-labeled iPS cells on gastric cancer *in vitro* and *in vivo*. (A) Hyperthermal effect of FMNP-labeled iPS cells treated under an external alternating magnetic field for 10 min. (Insets: bright-field microscopy image and infrared thermal images of FMNP-labeled iPS cells under the alternating magnetic field at various times.) (B) Hyperthermal effect of FMNP-labeled iPS cells on the gastric cancer model. [Insets: *ex vivo* tumor of the three groups of mice treated under different conditions. Test group, injected with FMNP-labeled iPS cells and external alternating magnetic field, average diameter: 5.2 mm; control group I, injected with FMNP-labeled iPS cells only; average diameter: 6.8 mm (*P*<0.05); control group II, without treatment; average diameter: 11.0 mm (*P*<0.01).] (C) Different hyperthermal effects of FMNP-labeled iPS cells on the *ex vivo* organs of tumor-free mice and tumor-bearing mice: (C_a_) infrared thermal image of the *ex vivo* tumor and organs of mouse models; (C_b_) thermal map obtained from the infrared thermal image by using IRS software; (C_c_) three-dimensional model based on the thermal map obtained by the IRS software. FMNP, fluorescent magnetic nanoparticle; iPS, induced pluripotent stem.

**Figure 6A** shows the time-temperature curve of FMNP-labeled iPS cells *in vitro*. The temperature of the labeled iPS cell pellets increased to 41.5 °C after 1 min of irradiation under an external alternating magnetic field. The internal temperatures of the labeled iPS cell pellets also increased with increasing time. The temperature increased to 53 °C after 2 min and increased to 62 °C after 4 min, and then maintained at this temperature with further increase in time.

To evaluate the potential of iPS cells for gastric cancer treatment, we injected FMNP-labeled iPS cells through the tail vein into gastric tumor-bearing mice, which were subjected under an external magnetic field. **Figure 6B** shows that the average diameter of the tumors was significantly smaller in the tumor-bearing group than that in the two control groups. The average tumor diameter in control group I, control group II, and treatment group was 6.8 mm (*P*<0.05), 11.0 mm (*P*<0.01), and less than 5.2 mm, respectively.

The survival ratio of tumor-bearing mice varied. The average survival time of mice in the control group II, control group I, and treatment group was 8, 12, and 15 weeks, respectively. These results showed that compared with the iPS cell therapy alone, hyperthermia markedly enhanced the inhibition of tumor cells and prolonged the survival of tumor-bearing mice.

Organs and tumor tissues were collected from the control and tumor-bearing groups to evaluate the effects of hyperthermia. The near infrared images of tumor and *ex vivo* organs were obtained and analyzed after treatment under an external alternating magnetic field for 5 min. **Figure 6C_a_** shows the infrared thermal images of the tumor and organs collected from euthanized mice. The grayscale intensity of the tumor site was strongest in tumor-bearing mice, where the core temperature was about 60 °C (**Figure 6C_b,c_**). The core temperature of the liver was less than 30 °C, which indicated that FMNP-labeled iPS cells entered into the tumor sites. Thus, the hyperthermal therapy could be performed using this method. Moderate temperatures (~30 °C) in the other organs were considered safe in the tumor-bearing mice. However, in the tumor-free control group, the strongest infrared thermal signal was located in the liver, whose core temperature reached 62 °C, followed by the lung with a core temperature of ~30 °C.

## Discussion

FMNP-labeled iPS cells can actively target gastric cancer cells *in vivo* and inhibit the growth of gastric cancer cells *in vitro*. Combined with treatment under an external magnetic field, FMNP-labeled iPS cells can be used for synergistic therapy for gastric cancer cells *in vitro*. FMNP-labeled iPS cells also can be used for therapy of other tumor cells *in vivo*. Nevertheless, the underlying mechanisms need to be verified.

FMNPs presents two interesting features, namely, fluorescence and superparamagnetism, which render these particles to be suitable for various applications, such as in bio-labeling, tracking of stem cell migration, biological separation, target imaging, drug delivery, and localized hyperthermia of tumors^35^^,^^36^. More importantly, FMNPs with positive surface charges can easily attach to the surface of cells via electrostatic adsorption, thereby inducing cellular endocytosis^37^^,^^38^. Our results also confirmed that the prepared FMNPs exhibit good biocompatibility and are thus suitable for clinical applications.

Regarding the mechanism of FMNP-labeled iPS cells in treating gastric cancer *in vitro*, we hypothesize that iPS cells secreted several specific anti-cancer factors into the culture medium; these factors can directly kill gastric cancer cells or regulate their growth by activating or inhibiting several specific pathways. Although the existence of this phenomenon was confirmed by our results, the identity of these factors and their functions remain unknown. Further work is therefore necessary.

The mechanism of FMNP-labeled iPS cells targeting gastric cancer cells *in vivo* includes the secretion of cytokines, chemokines, and/or growth factors into the blood or lymph circulation, which serve as candidate migration stimulatory signals. Some receptors on the iPS cell surface or iPS cells may secrete cytokines and chemokines, which combine with factors secreted by tumor cells; this phenomenon induces iPS cells to move to the tumor sites and is similar to the tropism of MSCs^32^. Nevertheless, the underlying molecular mechanism must be elucidated.

As iPS cells can actively target and recognize gastric cancer cells *in vivo* and may localize around the tumor site, FMNPs inside iPS cells can also localize around the tumor cells. FMNPs can produce heat energy when subjected under external magnetic field; in tumor sites, a high temperature of 62 °C was generated and this temperature can kill and inhibit the growth of tumor cells. However, in important organs, such as liver or kidney or lung, a temperature of 30 °C was generated, which cannot damage important organs. Therefore, the prepared FMNPs-labeled iPS cells could achieve the cancer therapy effect, which presents good clinical application prospects. The therapeutic effects of FMNP-labeled iPS cells coupled with the hyperthermal effects of FMNPs demonstrate a significant potential for clinical treatment of cancer.

**Figure 7:**
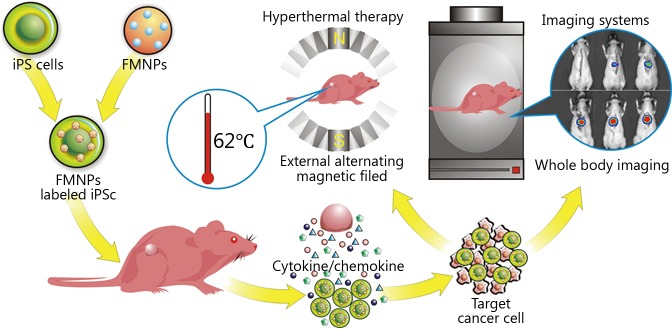
Schematic of the mechanism of FMNP-labeled human iPS cells for targeted imaging and therapy of tumor cells *in vivo*.

Furthermore, iPS cells inhibit cancer cell proliferation. The possible mechanism of iPS cells as a therapeutic agent for cancer mainly focuses on the following aspects: (I) iPS cells could produce numerous functional cells in tumor sites, and these cells can establish a normal relationship to tumor cells, deliver inhibitory factors to the tumor cells, and consequently inhibit tumor cell proliferation^39^^,^^40^. (II) iPS cells could regenerate different kinds of immunocytes, such as NK cells, activated macrophages, and cytolytic T cells. These regenerated cells could supply immunocytes into the tumor-bearing body and kill tumor cells^41^^,^^42^. (III) New functional cells produced by iPS cells could improve the microenvironment of the body and suppress cancer development^43^. Meanwhile, high concentrations of the differentiated signals could inhibit tumor cell growth. (IV) iPS cells could also regenerate new functional cells, which can restore immunity and quickly revive senescent cells, thereby preventing the occurrence of cancer^17^. Our results confirmed that FMNP-labeled iPS cells can be used for the synergistic therapy for *in vivo* tumor cells, such as gastric cancer. Nevertheless, the identity of the secreted factors and their functions remain unknown. The potential mechanism is shown in **Figure 7**. High performance FMNP-labeled human iPS cells exhibit considerable potential in various applications, such as dual-mode imaging and synergistic therapy of early gastric cancer *in vivo*. Future work should focus on the molecular mechanism of FMNP-labeled iPS in targeting and treating tumors.
